# Short Graphic Values History Tool for decision making during serious illness

**DOI:** 10.1136/bmjspcare-2018-001698

**Published:** 2019-02-06

**Authors:** John J You, Peter Allatt, Michelle Howard, Carole A Robinson, Jessica Simon, Rebecca Sudore, Amy Tan, Carrie Bernard, Marilyn Swinton, Xuran Jiang, Doug Klein, Michael McKenzie, Gillian Fyles, Daren Keith Heyland

**Affiliations:** 1 Medicine, McMaster University, Hamilton, Canada; 2 Bridgepoint Active Healthcare, Toronto, Ontario, Canada; 3 Family Medicine, McMaster University, Hamilton, Ontario, Canada; 4 School of Nursing, University of British Columbia Okanagan, Kelowna, British Columbia, Canada; 5 Oncology and Community Health Sciences, University of Calgary Cumming School of Medicine, Calgary, Alberta, Canada; 6 Division of Geriatrics, University of California, San Francisco, California, USA; 7 Department of Family Medicine, University of Calgary Cumming School of Medicine, Calgary, Alberta, Canada; 8 Department of Family Medicine, McMaster University, Hamilton, Ontario, Canada; 9 Department of Family and Community Medicine, University of Toronto, Toronto, Ontario, Canada; 10 Clinical Epidemiology and Biostatistics, McMaster University, Hamilton, Ontario, Canada; 11 Clinical Evaluation Research Unit (CERU), Kingston General Hospital, Kingston, Ontario, Canada; 12 Department of Family Medicine, University of Alberta, Edmonton, Alberta, Canada; 13 BC Cancer Agency, Vancouver, British Columbia, Canada; 14 BC Centre for Palliative Care, Vancouver, British Columbia, Canada; 15 Department of Critical Care Medicine, Queen's University, Kingston, Ontario, Canada

**Keywords:** values, communication, clinical decisions, questionnaire, validation

## Abstract

**Objectives:**

To develop and validate a values clarification tool, the Short Graphic Values History Tool (GVHT), designed to support person-centred decision making during serious illness.

**Methods:**

The development phase included input from experts and laypersons and assessed acceptability with patients/family members. In the validation phase, we recruited additional participants into a before–after study. Our primary validation hypothesis was that the tool would reduce scores on the Decisional Conflict Scale (DCS) at 1–2 weeks of follow-up. Our secondary validation hypotheses were that the tool would improve values clarity (reduce scores) more than other DCS subscales and increase engagement in advance care planning (ACP) processes related to identification and discussion of one’s values.

**Results:**

In the development phase, the tool received positive overall ratings from 22 patients/family members in hospital (mean score 4.3; 1=very poor; 5=very good) and family practice (mean score 4.5) settings. In the validation phase, we enrolled 157 patients (mean age 71.8 years) from family practice, cancer clinic and hospital settings. After tool completion, decisional conflict decreased (−6.7 points, 95% CI −11.1 to −2.3, p=0.003; 0–100 scale; N=100), with the most improvement seen in the values clarity subscale (−10.0 points, 95% CI −17.3 to −2.7, p=0.008; N=100), and the ACP-Values process score increased (+0.4 points, 95% CI 0.2 to 0.6, p=0.001; 1–5 scale; N=61).

**Conclusions:**

The Short GVHT is acceptable to end users and has some measure of validity. Further study to evaluate its impact on decision making during serious illness is warranted.

## Introduction

A clear understanding of patients’ values by substitute decision makers, clinicians and the patients themselves is critical to high-quality, person-centred decision making during serious illness.^1 2^ However, patients with serious illness are sometimes unclear about their own values or make value statements that are internally inconsistent. Substitute decision makers and clinicians too are often unaware of patients’ values.^3–5^ Without sound knowledge of patients’ values, care during serious illness may not be concordant with their values and goals, and patients may receive unwanted aggressive interventions.^6 7^

In this paper, we define values as things that are important to an individual in the context of medical decision making.^8^ By helping individuals identify and consider values in a more systematic way, values clarification tools may support better advance care planning (ACP) conversations between patients and their future substitute decision maker(s) or better goals-of-care conversations between clinicians, patients and substitute decision makers during serious illness.^2 9^ Although many values clarification tools have been previously developed, they have typically been created to address illness-specific medical or screening decisions.^10^ In contrast, the purpose of ACP and goals-of-care discussions is to inform a wide array of future decisions in the context of any serious life-limiting illness. Although some generic values history tools do exist, their development is not well described, they are not validated, or they may be too difficult or complex to be used by individuals with lower literacy or lower health literacy.^11–13^ Recently, to address these limitations, the Graphic Values History Tool (GVHT) was developed to be an easy to use, disease-agnostic tool to support better ACP between patients and substitute decision makers. Based on end-user feedback from patients and laypersons during its development, the GVHT includes graphic elements to increase clarity, including images to portray different health states.^14^

In September 2014, the Canadian Researchers at End of Life Network hosted a protocol development meeting focused on opportunities to test tools aimed at improving communication and decision making during serious illness. At this meeting, 20 investigators with expertise in serious illness communication and decision making, clinical ethics, palliative care, critical care, general internal medicine, questionnaire development, and health research methods reviewed the original GVHT. There was consensus that this tool could be useful in supporting serious illness communication and decision making in a broad range of clinical settings. However, investigators voiced concern about the length of the tool if it were to be implemented in clinical settings and recommended the development of a shorter version for this purpose. Accordingly, the objective of this study was to develop and validate an abridged version of the original GVHT, called the Short Graphic Values History Tool.

## Methods

### Design

This study consisted of two phases: a developmental phase and a validation phase.

### Setting and participants

To maximise generalisability of the findings, for both phases, we recruited patients from a variety of settings. In the family practice setting, patients were eligible if they were 50 years of age or older. In the oncology setting, patients were eligible if they were 18 years of age or older and had a diagnosis of cancer. In the hospital setting, patients were eligible if they were (1) 80 years of age or older, (2) 55 years of age or older with a chronic illness, such as congestive heart failure or chronic obstructive pulmonary disease at an advanced stage (see online Supplementary Appendix 1 for detailed clinical criteria), or (3) if a member of the healthcare team would not be surprised if the patient died in the next year. In all three settings, patients were excluded if they could not speak, read or write in English, or were unable to understand or participate in the study due to cognitive impairment (clinically assessed by research staff) or other medical reasons (eg, too unwell, hearing impairment, visual impairment and so on). If a hospitalised patient met the inclusion criteria but did not participate, we approached family members for enrolment instead, defined as individuals who knew the patient best, inclusive of partners, significant others and close friends (but excluding paid caregivers), and who had visited the patient in the hospital at least once. Family members were excluded if they were unable to communicate due to cognitive impairment or if they did not speak or read English. Participating family members were asked to complete the Short GVHT to reflect the patient’s values and not their own.

10.1136/bmjspcare-2018-001698.supp1 Supplementary data

#### Phase 1: development of the Short GVHT

The development of the original, longer GVHT, and demonstration of its face and content validity, is reported in detail elsewhere.^14^ The original tool (online supplementary appendix 2) has both a patient and substitute decision-maker version, and was developed based on literature review, input from content experts, laypersons and patients, and comprised 52 questions in 8 sections: quality of life—independence (eg, being able to go outside, needing help with self-care); quality of life—medical condition (eg, pain, breathing problems, cognitive deficits); value conflicts—part 1; value conflicts—part 2; are some conditions worse than death?; how do you weigh chances of survival?; impact of decisions on others; and religious, spiritual and cultural beliefs. The tool presents items on a 5-point Likert scale with colour shading, that is, 1=totally unacceptable (red), 5=totally acceptable (green). There was a free-text box after each question to allow the respondent to further elaborate on their response. One section (values conflicts—part 1) assessed value conflicts using a line with two anchors to depict trade-offs between different values (eg, quality and quantity of life). The tool contained graphic elements to increase clarity, including images to portray the concept (eg, being wheelchair users) beside each question.

The development of the Short GVHT was an iterative process. Based on input from content experts who attended the September 2014 protocol development meeting and further deliberation of a study steering committee (PA, JJY, MS and DKH), items were retained, combined or removed based on their face and content validity to produce an initial draft of a Short GVHT comprising five rather than eight sections: (1) acceptable quality of life; (2) value conflicts; (3) are some conditions worse than death?; (4) impact of decision on others; and (5) religious, spiritual and cultural beliefs. In March 2015, we then asked 20 laypersons of varying age (range 20–95 years), education (grade school to postgraduate) and health status (healthy to multiple comorbidities) to review the draft version of the tool and provide comments, written directly on the tool or by email. Based on this feedback, we did not add or remove any items, but made 3 formatting changes and 11 wording revisions (see online supplementary appendix 3) to create final versions (patient and substitute decision maker) of the Short GVHT (full name: ‘What’s Important to Me: Graphic Values History Tool’) consisting of 32 questions in 5 sections (online supplementary appendix 4).

For the final step of phase 1, we obtained patient and family member ratings of the acceptability and sensibility of the Short GVHT. To do this, we enrolled a convenience sample of participants from a family practice in Ontario, a family practice in Alberta and a hospital medical ward (Foothills Medical Centre, Calgary, Alberta) using the eligibility criteria described above. Participants completed the Short GVHT, then completed a questionnaire (see table 1) that was informed by the framework for evaluation of sensibility developed by Feinstein, and included items from other instruments that have also measured these constructs.^15–18^

**Table 1:** Acceptability of the Short Graphic Values History Tool

Item	Hospital (n=10)	Family practices (n=12)
Clarity of language (1=very unclear; 5=very clear)	4.4 (0.5)	4.5 (0.5)
Amount of information (1=much less than I wanted; 5=much more than I wanted)	3.4 (0.7)	3.0 (0.0)
Ease of use (1=very difficult; 5=very easy)	4.2 (0.4)	4.1 (0.9)
Helpfulness for a person thinking about medical treatment for a serious illness (1=very unhelpful; 5=very helpful)	4.5 (0.5)	4.8 (0.5)
Likely to use if recommended by doctor (1=definitely would not; 5=definitely would)	4.4 (0.8)	4.3 (0.7)
Recommend to someone else for the purpose of discussing options for medical treatment of a serious illness (1=definitely would not; 5=definitely would)	4.2 (1.0)	3.8 (1.2)
Global rating of tool: ‘Overall, how would you rate the tool?’ (1=very poor; 5=very good)	4.3 (0.8)	4.5 (0.5)

All data are reported as mean (SD).

#### Phase 2: validation phase

For the validation phase, we recruited participants from 12 family medicine practices in the provinces of Ontario (n=9), Alberta (n=1) and British Columbia (n=2), 2 oncology centres in British Columbia, and medical wards of 2 teaching hospitals in Calgary, Alberta (Foothills Medical Centre) and Hamilton, Ontario (Hamilton General Hospital), all in Canada. Sites used different recruitment procedures depending on the clinical setting. In the family practice and oncology clinic settings, physicians identified eligible patients, and research assistants approached interested patients in person at clinic appointments or by telephone for their consent to participate in the study. In the hospital setting, eligible patients were identified by screening medical records of patients who had been admitted to the medical ward for 2–7 days. Bedside nurses approached eligible patients to introduce the study, and consent to participate in the study was obtained by the research assistant.

Baseline questionnaires administered in person by research assistants included participants’ demographic information, preferences for use or non-use of life-sustaining treatments, the low-literacy version of the Decisional Conflict Scale (primary outcome) with respect to the above preference about life-sustaining treatments,^19 20^ and the values domain of the validated Advance Care Planning Engagement Survey (ACP-Values).^21^

The low-literacy version of the Decisional Conflict Scale (online supplementary appendix 5) is a 10-item validated instrument consisting of four subscales that captures personal perceptions of feeling: (1) uncertain about a treatment choice (uncertainty subscale), (2) uninformed about choosing treatment options (informed subscale), (3) unclear about personal values (values clarity subscale), and (4) supported in decision making (support subscale). The overall scale and each subscale are each scored from 0 to 100, with lower scores corresponding to more desirable scores, that is, less decisional conflict, more certain about best choice, more informed, more clarity about personal values and more supported in decision making, respectively.

The ACP Engagement Survey is a validated instrument that covers four domains of ACP behaviours related to surrogate decision makers, values (quality of life), flexibility for surrogates and asking doctors questions. Each domain has been validated for individual use. For this study, we used the 22-item values domain (ACP-Values) which focuses on the identification and discussion of one’s values (online supplementary appendix 6). The instrument includes both 14 ‘Process’ items of knowledge, contemplation, self-efficacy and readiness assessed on Likert scales from 1 to 5 points, and 8 ACP ‘Action’ items using binary (yes/no) response options. Accordingly, the ACP-Values scale produces both a process score (average of Likert scale responses from 1 to 5) and an action score (total of yes/no responses from 0 to 8).

In the oncology and family practice settings, after collection of baseline measures, participants were introduced to the Short GVHT by research staff and asked to complete the tool at home. Two weeks after enrolment, we contacted the participants by telephone to readminister the low-literacy Decisional Conflict Scale and the ACP-Values scale. In the hospital setting, after collection of baseline measures, research staff introduced the Short GVHT and recorded participants’ responses to the tool. After 1 week, we readministered the low-literacy Decisional Conflict Scale and the ACP-Values scale. Because the primary aim of this phase of the study was to evaluate construct validity (ie, this was not a study of clinical effectiveness), we wanted to focus on measuring very proximal perceptions of participants after completing the tool. For this reason, we elected to use a relatively short follow-up time of 1–2 weeks; however, before considering participants lost to follow-up, we made several attempts to contact participants for up to 6 weeks postenrolment.

### Statistical analysis

We describe the ratings of acceptability and sensibility and the baseline characteristics of participants using counts and proportions for categorical variables, and means, SD and ranges for continuous variables; we excluded cases from these analyses if they had missing data for 50% or more of baseline items.

For the validation phase, we assessed construct validity of the Short GVHT by testing a series of hypotheses that would provide evidence that the tool does what it is intended to do. Our primary validation hypothesis was that the tool would be associated with a reduction in decisional conflict. Our secondary validation hypotheses were that (1) the tool would improve the values clarity subscale of the Decisional Conflict Scale more than other subscales; and (2) the tool would increase engagement in ACP processes related to identification and discussion of one’s values (ACP-Values process scores), but not necessarily lead to a change in ACP-Values action scores, since our study procedures did not include explicit steps to encourage participants or clinicians to take further action. To test these hypotheses, we used paired t-tests to compare scores on outcome measures of interest (eg, Decisional Conflict Scale and its subscales, ACP-Values process and action scores) before versus after completion of the Short GVHT. We reported the change in scores as absolute differences and 95% CI. We used a complete case analysis approach for these analyses; that is, we only included cases with non-missing data for items before and after completion of the Short GVHT. We calculated that a sample size of 120 evaluable participants would provide 90% power to detect an effect size as small as 0.3 in the change in decisional conflict from baseline to follow-up. We used the SAS V.9.4 statistical package to conduct the analyses. A p value of 0.05 was considered statistically significant.

## Results

### Phase 1: acceptability and sensibility of the Short GVHT

To formally assess the acceptability and sensibility of the tool, we enrolled a convenience sample of 10 participants (5 patients, age 77.8±9.6 years, 40% female; 5 family members, age 66.2±17.7, 100% female) from a hospital medical ward and 12 patients (age 66.4±7.7 years, 75% female) from a family practice setting. Participants gave the tool positive ratings, including global rating scores of 4.3±0.8 and 4.5±0.5 from hospital and family practice participants, respectively (1=very poor; 5=very good) (table 1).

### Phase 2: validation of the Short GVHT

Between July 2015 and August 2016, we approached 560 eligible patients from family practice, cancer clinic and hospital settings. Of these, 403 did not participate, resulting in a study cohort of 157 participants (participation rate of 28%). Of the 157 participants, 12 patients did not complete all baseline measures (ie, defined as greater than or equal to 50% of questions having missing data), resulting in an evaluable cohort of 145 participants (table 2).

**Table 2:** Baseline characteristics of study population for the validation phase

	Patients N=145
Age, years, mean±SD (range)	71.8±12.6 (21.8–101.0)
Sex	
Male	74 (51.0%)
Female	68 (46.9%)
Missing	3 (2.1%)
Ethnicity	
Caucasian	130 (89.7%)
Asian	2 (1.4%)
East Indian	5 (3.4%)
First Nations/Inuit/Metis or Aboriginal	1 (0.7%)
African/Black North American	2 (1.4%)
Other	2 (1.4%)
Missing	3 (2.1%)
Formal religious group	
Protestant	51 (35.2%)
Catholic	30 (20.7%)
Jewish	1 (0.7%)
Muslim	1 (0.7%)
None	40 (27.6%)
Other	17 (11.7%)
Missing	5 (3.4%)
Highest level of education	
Did not complete high school	36 (24.8%)
Completed high school	28 (19.3%)
Some university education or completed other postsecondary programme	51 (35.2%)
University undergraduate degree	18 (12.4%)
University graduate degree	10 (6.9%)
Missing	2 (1.4%)
Place of residence in the past month	
Own home	131 (90.3%)
Retirement residence	7 (4.8%)
Long-term care home	2 (1.4%)
Hospital	1 (0.7%)
Other	2 (1.4%)
Missing	2 (1.4%)
Comorbidities*	
Heart disease	43 (29.7%)
High blood pressure	78 (53.8%)
Lung disease	24 (16.6%)
Diabetes	32 (22.1%)
Ulcer or stomach disease	37 (25.5%)
Kidney disease	18 (12.4%)
Liver disease	15 (10.3%)
Anaemia or other blood disease	26 (17.9%)
Cancer	70 (48.3%)
Depression	40 (27.6%)
Osteoarthritis, degenerative arthritis	0 (0.0%)
Back pain	71 (49.0%)
Rheumatoid arthritis	16 (11.0%)
Number of comorbidities per patient, mean±SD (range)	3.8±2.1 (0.0–10.0)
Frailty†	
Very fit	18 (12.4%)
Well	24 (16.6%)
Managing well	44 (30.3%)
Vulnerable	39 (26.9%)
Mildly frail	12 (8.3%)
Moderately frail	4 (2.8%)
Severely frail	2 (1.4%)
Very severely frail	0 (0.0%)
Missing	2 (1.4%)
Quality of life*	
Excellent	21 (14.5%)
Very good	42 (29.0%)
Good	41 (28.3%)
Fair	32 (22.1%)
Poor	8 (5.5%)
Missing	1 (0.7%)

*Based on patient self-report.

†Based on patient self-report using the Clinical Frailty Scale.^24^

#### Decisional Conflict Scale

Baseline and follow-up scores on the Decisional Conflict Scale and its subscales for all 100 patients with non-missing data for both time points are reported in table 3. The total score at baseline was 25.0±26.1 (mean±SD) out of a maximum score of 100 (0=no decisional conflict; 100=extremely high decisional conflict). After completion of the Short GVHT, there was a statistically significant decrease in the total score compared with baseline (decrease of 6.7 points, 95% CI −11.1 to −2.3, p=0.003). Of the four subscales (0=desirable; 100=undesirable) of the Decisional Conflict Scale, the largest decrease was in the values clarity subscale (ie, respondents had greater clarity about their personal values), which decreased by 10.0 points (95% CI −17.3 to −2.7, p=0.008). This is in contrast to smaller changes in the uncertainty subscale (decrease of 8.5 points, 95% CI −14.6 to −2.4, p=0.007), informed subscale (decrease of 8.0 points, 95% CI −14.8 to −1.2, p=0.02) and support subscale (decrease of 2.0 points, 95% CI −5.7 to 1.7, p=0.29) (table 3).

**Table 3:** Decisional Conflict Scale before and after completion of the Short Graphic Values History Tool

	n	Before Mean±SD	After Mean±SD	Change (95% CI)	P value
Total score	100	25.0±26.1	18.7±20.3	−6.7 (−11.1 to −2.3)	0.003
Uncertainty subscore	100	19.4±31.9	11.3±26.2	−8.5 (−14.6 to −2.4)	0.007
Informed subscore	100	38.2±38.0	31.0±31.4	−8.0 (−14.8 to −1.2)	0.02
Values clarity subscore	100	27.9±36.1	18.4±29.0	−10.0 (−17.3 to −2.7)	0.008
Support subscore	100	13.4±20.2	11.6±19.5	−2.0 (−5.7 to 1.7)	0.29

Total score: 0 (no decisional conflict) to 100 (extremely high decisional conflict); subscales: 0 (‘good’, eg, ‘extremely certain’) to 100 (’bad’, eg, ’extremely uncertain’).

#### ACP Engagement Survey: values domain (ACP-Values)

Baseline and follow-up scores on the ACP-Values instrument for all 61 patients with non-missing data for both time points are reported in table 4. At baseline, the ACP-Values process score was 3.3±0.9 out of a maximum possible score of 5, and the ACP-Values action score was 2.6±2.9 out of a maximum possible score of 8. After completion of the Short GVHT, there was a statistically significant increase in the ACP-Values process score compared with baseline (+0.4 points, 95% CI 0.2 to 0.6, p=0.001), but no statistically significant change in the ACP-Values action score (+0.2 points, 95% CI −0.4 to 0.8, p=0.58) (table 4). The increase in the ACP-Values process score was driven by increases in the contemplation (+0.4 points, 95% CI 0.2 to 0.6, p=0.0001) and readiness (+0.5 points, 95% CI 0.1 to 1.0, p=0.01) components of the score.

**Table 4:** Values domain of Advance Care Planning Engagement Survey (ACP-Values) before and after completion of the Short Graphic Values History Tool

	n	Before Mean±SD	After Mean±SD	Change (95% CI)	P value
Process score*	61	3.3±0.9	3.7±0.9	0.4 (0.2 to 0.6)	0.001
Contemplation	61	2.8±1.1	3.2±1.0	0.4 (0.2 to 0.6)	0.0001
Self-efficacy	61	4.3±0.8	4.3±0.8	0.0 (−0.2 to 0.2)	0.90
Readiness	61	3.1±1.2	3.7±1.7	0.5 (0.1 to 1.0)	0.01
Action score†	61	2.6±2.9	2.8±3.0	0.2 (−0.4 to 0.8)	0.58
Health situations	61	1.3±1.5	1.3±1.5	0.0 (−0.3 to 0.3)	0.92
Medical care	61	1.3±1.5	1.5±1.6	0.2 (−0.2 to 0.5)	0.29

*Process score: 1–5.

†Action score: 0–8.

## Discussion

We have developed the Short GVHT, a disease-agnostic values clarification tool designed to support person-centred decision making during serious illness. Our findings demonstrate that the tool has face and content validity and good acceptability based on the input of content experts and laypersons during its development and field testing with patients and family members in outpatient and inpatient settings. Our study also provides some evidence of construct validity of the Short GVHT based on the results of our before–after evaluation.

In particular, our results were consistent with our primary validation hypothesis that the Short GVHT would be associated with a reduction in decisional conflict. Furthermore, because the Short GVHT is a values clarification tool, we anticipated that the values clarity subscale of the Decisional Conflict Scale would show the most improvement compared with other subscales. Finally, we hypothesised that the Short GVHT would be associated with an increase in the ACP-Values process score (ie, constructs of contemplation, self-efficacy and readiness), but not necessarily a change in actions related to the identification or discussion of values. This was because our study protocol did not include a follow-up visit with their clinician to discuss their responses to the Short GVHT, or any other explicit actions for participants or clinicians to take related to the discussion of their values. Given that our findings were consistent with our validation hypotheses, we conclude that the Short GVHT has some evidence for construct validity.

Our study adds to the literature on values clarification tools. First, many existing values clarification tools are too narrowly focused on specific medical decisions (eg, in cancer or reproductive care) to be broadly applicable to decision making during serious illness.^10^ In contrast, since the Short GVHT is a disease-agnostic tool, it can be helpful in supporting the wide range of decisions that may be encountered during serious illness. Second, although some generic values history tools have been previously developed, they have limitations. For instance, in the late 1980s and early 1990s, Justin,^11^ Lambert *et al*,^12^ and Doukas and McCullough^13^ each described their own values history tools intended to inform future decisions about life-sustaining treatments. Although similar in nature to the Short GVHT, the development of these tools was not well described and none of these tools were evaluated for their validity in supporting communication or decision making during serious illness. Our study addresses these limitations by describing the development of the Short GVHT in detail, asking end users to assess its acceptability and sensibility, and evaluating the validity of the tool in a before–after study.

Our evaluation of construct validity was focused on measuring very proximal perceptions of participants after completing the tool. Accordingly, the magnitude of effect that we observed was small and we did not observe an increase in ACP-related actions. This was anticipated due to the relatively short follow-up time, which may not have allowed sufficient time for participants to complete these actions and because our intervention did not include explicit supports or prompts to patients, substitute decision makers or clinicians to discuss values during follow-up. Future research could focus on the development and evaluation of clinician communication strategies to leverage the information gleaned from patients or substitute decision makers using the Short GVHT. For example, an unanswered question is whether values clarification methods should precede clinical encounters, be used during clinical encounters or follow these conversations.^22^ Finally, future research could include the conduct of prospective studies that evaluate the effect of the Short GVHT on the quality of actual medical decisions that are made during future episodes of serious illness.

The strengths of our study include the involvement of a wide range of individuals, including end users, in the development of the Short GVHT. In addition, we used validated instruments to prospectively assess efficacy and construct validity and conducted this work in several different clinical settings to enhance the credibility and applicability of our findings. Finally, we adhered to best practices in the conduct and reporting of survey research.^23^ However, our study also has limitations. First, an appreciable proportion of participants who were approached did not participate or were lost to follow-up and had missing outcome data. To the extent that non-participating patients or patients with missing outcome data were systematically different from those who had completed the outcome assessments, this may introduce a risk of bias and limit the generalisability of our findings. In addition, our findings may not be applicable to more diverse populations who may have different approaches to engaging in decision making during serious illness. Second, the magnitude of effects that we observed was modest. However, this study was not an implementation study aimed at assessing the clinical effectiveness of the tool in clinical practice. Instead, our objective was to develop and validate the tool. Our findings of statistically significant and clinically sensible changes in constructs in response to the tool provide some evidence of the tool’s construct validity.

In conclusion, the Short GVHT is a disease-agnostic values clarification tool that has good acceptability, face validity, content validity, and some evidence of efficacy and construct validity. Further evaluation may be warranted to prospectively evaluate whether the tool can increase the quality of decision making during serious illness and improve concordance of care with patients’ values and goals.
