# Supplementary material for: Short Graphic Values History Tool for decision making during serious illness
Source: BMJ Support Palliat Care. 2019 Feb 6;12(e6):e777–84. doi: 10.1136/bmjspcare-2018-001698 (PMC9726972; doi:10.1136/bmjspcare-2018-001698)
Supplement: Supplementary data [file bmjspcare-2018-001698supp001.pdf]

Appendix 1. Detailed eligibility criteria for patients in the hospital setting

1. Patient is 80 years of age or older;

   OR

2. Patient is 55 years of age or older with one or more of the following advanced medical diagnoses:

- Chronic obstructive lung disease (2 or more of: baseline PaCO2 of ≥45 mm Hg, cor pulmonale, respiratory failure episode within the preceding year, forced expiratory volume in 1 sec ≤ 0.5 L);
- Congestive heart failure (New York Heart Association class IV symptoms and left ventricular ejection fraction ≤ 25%);
- Cirrhosis (confirmed by imaging studies or documentation of esophageal varices, plus one of three conditions: hepatic coma, Child's class C liver disease, or Child's class B liver disease with gastrointestinal bleeding);
- Cancer (metastatic cancer or stage IV lymphoma)
- End-stage dementia (inability to perform all activities of daily living, mutism or minimal verbal output secondary to dementia, bed-bound state prior to acute illness)
- Renal failure (chronic renal failure requiring dialysis)

   OR

3. Any patient for whom a clinical team member (e.g., attending physician or bedside nurse) would not be surprised if the patient died within the next 6 months

Appendix 2. Original Graphic Values History Tool (GVHT); patient version

Appendix J

ID# ____ - ____ . ____

Graphic Values History

Questions for Patients

Peter Allatt, Clinical Ethicist

Version Date: August 1, 2014

Values History Process

The values history process is made up of 4 steps.

Step 1. Patient

1. Patient chooses the format most comfortable completing the GVH – paper, laptop, tablet
2. Researcher
   a. Orients patient to the GVH.
   b. Instructs patient to complete the GVH based on his/her values.
3. Patient completes the Graphic Values History for patients, without SDM input.
   a. If necessary researcher provides assistance completing document
      i. Acting as a scribe
      ii. Answering questions.

Step 2. Substitute Decision Maker

1. SDM chooses the format most comfortable completing the GVH – paper, laptop, tablet
2. Researcher
   a. Orients SDM to the GVH.
   b. Instructs SDM to complete the GVH based on his/her understanding of the patient's values.
3. SDM completes the Graphic Values History for SDMs, without patient input.
   a. If necessary researcher provides assistance completing document
      i. Acting as a scribe
      ii. Answering SDM's questions.

Step 3. Facilitated Discussion

1. Researcher will facilitate discussion of GVH responses, comparing
   a. Patient responses
   b. SDM responses
2. Intent of the facilitated discussion is for:
   a. SDM to hear the patient's responses, and why
   b. Patient to hear SDM's responses, and why
   c. SDM to understand patient values
3. If appropriate/necessary GVH can be updated

4. Advise the patient and SDM to keep the GVH and update as necessary
5. Advise the SDM to bring the GVH to the hospital if the patient is admitted

Step 4. Decision Making

The subsitute decision maker's responsibility for decision making begins when the patient is no longer capable to make decisions and needs treatment decisions made. The doctor caring for the patient will make this determination. At this time the substitute decision maker should use the GVH to determine what the patient would want in the situation. Making decisions based on applicable wishes is what is required under the Health Care Consent Act and demonstrates respect for the patient.

Graphic Values History

Definition

A values history is a document that asks questions to help you think about **your** values and preferences. It helps you identify what is important to you. The completed document can help guide your substitute decision maker (SDM, e.g., POA, spouse) in the event you are not able to make decisions.

Introduction

This graphic values history is intended to assist you in thinking about your personal values.

There are eight sections to the graphic values history:

1. Quality of Life - Independence
2. Quality of Life - Medical condition
3. Value Conflicts - Part 1
4. Value Conflicts - Part 2
5. Are some conditions worse than death?
6. How do you weigh chances of survival?
7. Impact of decisions on others
8. Religious / Spiritual / Cultural Beliefs

Please take as much time as you need to complete this graphic values history. While adding comments takes time, this provides valuble information about what you were thinking. This will be helpful during the Values History Discussion. You are not required to answer every question; however, doing so will give useful information to your treatment team and to your substitute decision maker in the event that you are not able to make decisions for yourself.

Graphic Values History

Useage

This document can be used along with a “Living Will” or other statement of treatment wishes. In the event that you are found to be incapable of making treatment decisions, your substitute decision maker will be required to follow your previously expressed wishes which are applicable to the circumstances. This is why it is important to discuss your values by reviewing this document with your SDM.

If you change your mind, revise your values history and discuss the changes with your substitute decision maker

You JJ, *et al. BMJ Supportive & Palliative Care* 2022; 12:e777–e784. doi: 10.1136/bmjspcare-2018-001698

Graphic Values History

1. Quality of Life - Dependence - Independence†
Definition
Quality of life - The degree of satisfaction, comfort and enjoyment in life, what makes life worth living.

This section looks at the value of independence in **YOUR** quality of life. Consider whether your quality of life would be acceptable if you found yourself in the following circumstance, with **no reasonable chance of improvement.**

Instructions: Circle the most appropriate answer.

† Adapted from Scheunemann LP, Arnold RM, White DB. The facilitated values history: helping surrogates make authentic decisions for incapacitated patients with advanced illness. Am J Respir Crit Care Med 186(6). 15 Sept 2012

**What would I say about my quality of life if I were . . .**			**Totally Unacceptable**	**Somewhat Unacceptable**	**Uncertain**	**Somewhat Acceptable**	**Totally Acceptable**
a. Unable to walk, could only get around by wheelchair	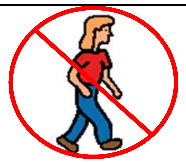	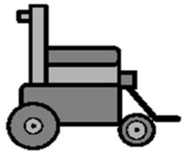	**1**	**2**	3	**4**	**5**
Comments							
b. Unable to feed myself	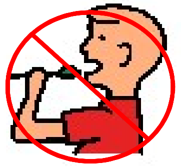	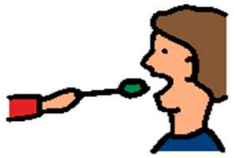	**1**	**2**	3	**4**	**5**
**Comments**							

You JJ, *et al. BMJ Supportive & Palliative Care* 2022; 12:e777–e784. doi: 10.1136/bmjspcare-2018-001698

Graphic Values History

**What would I say about my quality of life if I were . . .**			**Totally Unacceptable**	**Somewhat Unacceptable**	**Uncertain**	**Somewhat Acceptable**	**Totally Acceptable**
c. Unable to wash myself	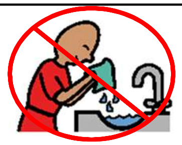	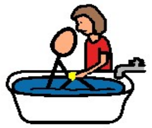	**1**	**2**	3	**4**	**5**
**Comments**							
d. Unable to make my own decisions	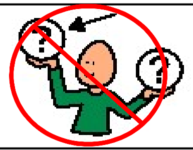		**1**	**2**	3	**4**	**5**
**Comments:**							
e. Not able to return to the place where I lived before I got sick	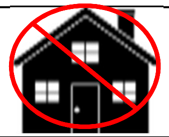		**1**	**2**	3	**4**	**5**
**Comments:**							

You JJ, *et al. BMJ Supportive & Palliative Care* 2022; 12:e777–e784. doi: 10.1136/bmjspcare-2018-001698

Graphic Values History

2. Quality of Life - State of Health

Definition

Quality of life - The degree of satisfaction, comfort and enjoyment in life, what makes life worth living.

This section looks at medical conditions that might change YOUR quality of life. Consider whether your quality of life would be acceptable if you found yourself in the following circumstance, with **no reasonable chance of improvement**.

Instructions: Circle the most appropriate answer.

**What would I say about my quality of life if I were . . .**		**Totally Unacceptable**	**Somewhat Unacceptable**	**Uncertain**	**Somewhat Acceptable**	**Totally Acceptable**
a. Unable to swallow safely	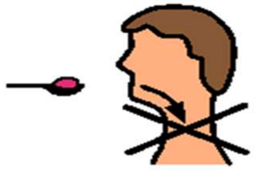	**1**	**2**	3	**4**	**5**
Comments						
b. Fed by tube into the stomach	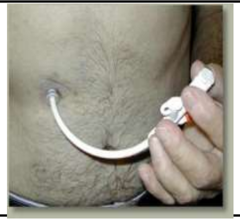	**1**	**2**	3	**4**	**5**
Comments						

Graphic Values History

**What would I say about my quality of life if I were . . .**		**Totally Unacceptable**	**Somewhat Unacceptable**	**Uncertain**	**Somewhat Acceptable**	**Totally Acceptable**
c. Unable to understand what others say (able to hear the voice or see print, but I can't make sense of the words)	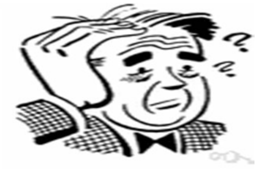	**1**	**2**	3	**4**	**5**
Comments						
d. Unable to communicate my thoughts (know what I want to say, but have trouble saying or writing)	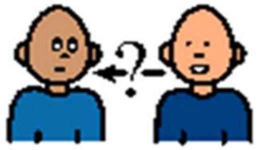	**1**	**2**	3	**4**	**5**
Comments:						
e. Unable to speak or understand speech and unable to read or write.	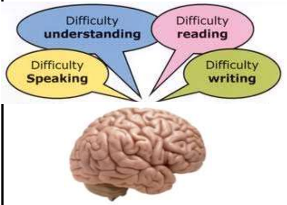	**1**	**2**	3	**4**	**5**
Comments:						

You JJ, *et al. BMJ Supportive & Palliative Care* 2022; 12:e777–e784. doi: 10.1136/bmjspcare-2018-001698

Graphic Values History

**What would I say about my quality of life if I were . . .**		**Totally Unacceptable**	**Somewhat Unacceptable**	**Uncertain**	**Somewhat Acceptable**	**Totally Acceptable**
f. In constant physical pain	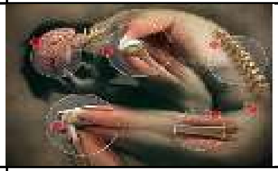	**1**	**2**	3	**4**	**5**
Comments:						
g. Constantly short of breath	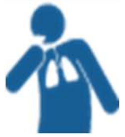	**1**	**2**	3	**4**	**5**
Comments:						
h. Constantly thirsty	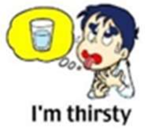	**1**	**2**	3	**4**	**5**
Comments:						
i. Constantly nauseated (feel like I'm going to throw up)	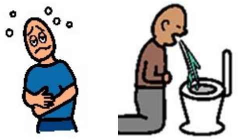	**1**	**2**	3	**4**	**5**
Comments:						

You JJ, *et al. BMJ Supportive & Palliative Care* 2022; 12:e777–e784. doi: 10.1136/bmjspcare-2018-001698

Graphic Values History

3. Questions exploring value conflicts (part 1) †
When two values conflict, which value takes priority (more important)?

Instructions: Circle the point on the **line** (range) that shows which value is most important to you.

† Adapted from Scheunemann LP, Arnold RM, White DB. The facilitated values history: helping surrogates make authentic decisions for incapacitated patients with advanced illness. Am J Respir Crit Care Med 186(6). 15 Sept 2012

a. What would I say is more important -- **quality of life** or **quantity of life**?

**Quality of Life**<br>How well I live					**Quantity of Life**<br>How long I live

Comments:

Graphic Values History

b. What would I say is more important -- **pain control** or **remaining clear minded?**

**Pain Control**					**Clear minded**
Free of pain, but possibly sleepy/confused					Able to think clearly, not sleepy but in pain

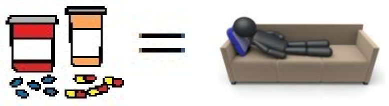

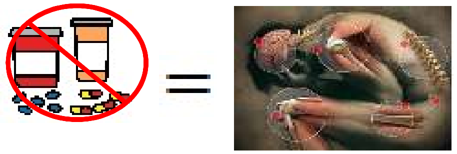

Comments:

c. What would I say is more important -- **symptom control** (nausea, anxiety) or **remaining clear minded?**

**Symptom Control**					**Clear minded**
Free of symptoms but possibly sleepy/confused					Able to think clearly, not sleepy but nauseated or anxious

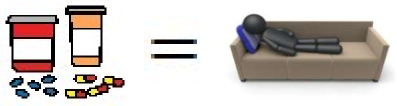

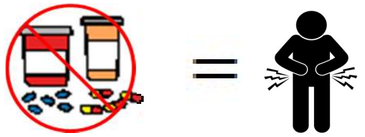

Comments:

You JJ, *et al. BMJ Supportive & Palliative Care* 2022; 12:e777–e784. doi: 10.1136/bmjspcare-2018-001698

4. Questions exploring value conflicts (part 2)

When two values conflict, which value takes priority (most important)? How do you assess quality of life under the following circumstances?

Instructions: Circle the most appropriate answer.

a. What would I say if my illness meant that I needed assistance with basic physical care (e.g., toileting, washing, feeding), but was able to think clearly and communicate with my family?

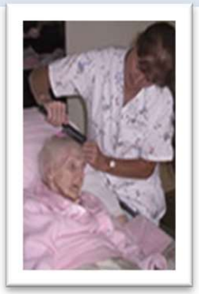

Totally Unacceptable	Somewhat Unacceptable	Uncertain	Somewhat Acceptable	Totally Acceptable
1	2	3	4	5

Comments:

b. What would I say if I were no longer able to make decisions for myself or look after myself, but I could recognize family?

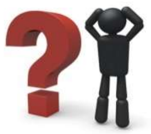

Totally Unacceptable	Somewhat Unacceptable	Uncertain	Somewhat Acceptable	Totally Acceptable
1	2	3	4	5

Comments:

Graphic Values History

c. What would I say if I were no longer able to make decisions for myself or look after myself, **and** was **not awake** or able to recognize my family?

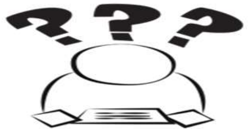

Totally Unacceptable	Somewhat Unacceptable	Uncertain	Somewhat Acceptable	Totally Acceptable
1	2	3	4	5

Comments:

d. Would I say it was worth going through invasive treatment to live **one more day**?

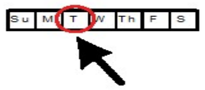

Totally Unacceptable	Somewhat Unacceptable	Uncertain	Somewhat Acceptable	Totally Acceptable
1	2	3	4	5

Comments:

e. Would I say it was worth going through invasive treatment to live **one more month**?

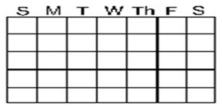

Totally Unacceptable	Somewhat Unacceptable	Uncertain	Somewhat Acceptable	Totally Acceptable
1	2	3	4	5

Comments:

Invasive - a treatment that involves putting something into the body or cutting into the body

You JJ, *et al. BMJ Supportive & Palliative Care* 2022; 12:e777–e784. doi: 10.1136/bmjspcare-2018-001698

Graphic Values History

5. Are some conditions worse than death? ‡

Many treatments keep people alive even if there is no reasonable chance that the treatment will reverse or improve their condition. This worksheet looks at such situations.

In each situation described below, ask yourself: what would I want if the treatment would **NOT reverse or improve my condition**?

Instructions: Circle the most appropriate answer.

‡Adapted from American Bar Association Commission on Law and Aging. Consumer's Tool Kit For Health Care Advance Care Planning: Tool #2. Are some conditions worse than death? Second edition, 2005

**What if I . . .**		**Definitely do not want treatment that prolongs life**	**May not want treatment that prolongs life**	**Uncertain**	**May want treatment that prolongs life**	**Definitely want treatment that prolongs life**
a. Could no longer recognize (know) or interact (talk or do things with) with family or friends.	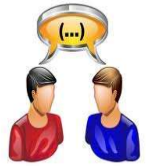	**1**	**2**	3	**4**	**5**
Comments						

Graphic Values History

**What if I . . .**		**Definitely do not want treatment that prolongs life**	**May not want treatment that prolongs life**	**Uncertain**	**May want treatment that prolongs life**	**Definitely want treatment that prolongs life**
b. Could no longer think or speak clearly.	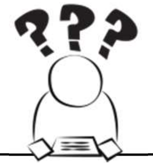	**1**	**2**	3	**4**	**5**
Comments						
c. Could no longer respond to commands or requests.	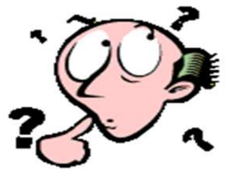	**1**	**2**	3	**4**	**5**
Comments						
d. Could no longer walk, but could get around in a wheelchair.	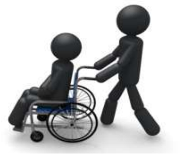	**1**	**2**	3	**4**	**5**
Comments						

Graphic Values History

**What if I . . .**		**Definitely do not want treatment that prolongs life**	**May not want treatment that prolongs life**	**Uncertain**	**May want treatment that prolongs life**	**Definitely want treatment that prolongs life**
e. Could no longer get outside and must spend all day at home.	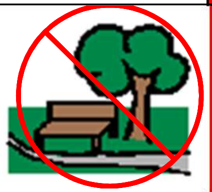	**1**	**2**	3	**4**	**5**
Comments						
f. Was in severe untreatable physical pain most of the time.	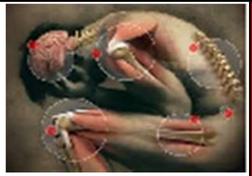	**1**	**2**	3	**4**	**5**
Comments						
g. Was in severe discomfort (such as nausea, diarrhea) most of the time .	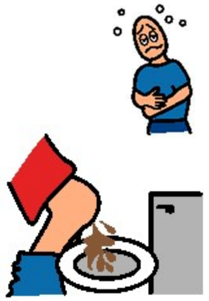	**1**	**2**	3	**4**	**5**
Comments						

You JJ, *et al. BMJ Supportive & Palliative Care* 2022; 12:e777–e784. doi: 10.1136/bmjspcare-2018-001698

Graphic Values History

**What if I . . .**		**Definitely do not want treatment that prolongs life**	**May not want treatment that prolongs life**	**Uncertain**	**May want treatment that prolongs life**	**Definitely want treatment that prolongs life**
h. Needed a feeding tube to stay alive.	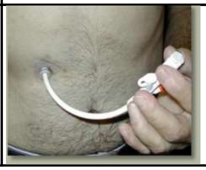	**1**	**2**	3	**4**	**5**
Comments						
i. Need to be connected to a machine 4 - 6 hours per treatment, 3 treatments per week. Treatments clean my blood and remove fluid	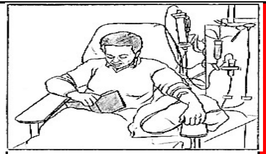	**1**	**2**	3	**4**	**5**
Comments						
j. Need to be connected to a breathing machine 24 hrs a day in order to breathe	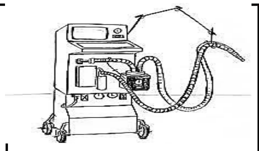	**1**	**2**	3	**4**	**5**
Comments						

You JJ, *et al. BMJ Supportive & Palliative Care* 2022; 12:e777–e784. doi: 10.1136/bmjspcare-2018-001698

Graphic Values History

**What if I . . .**		**Definitely do not want treatment that prolongs life**	**May not want treatment that prolongs life**	**Uncertain**	**May want treatment that prolongs life**	**Definitely want treatment that prolongs life**
k. Needed someone to take care of me 24 hours a day.	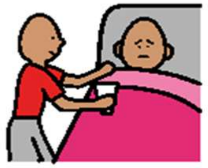	**1**	**2**	3	**4**	**5**
Comments						
l. Could no longer control my bladder (may need to wear adult diapers).	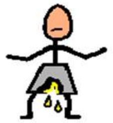	**1**	**2**	3	**4**	**5**
Comments						
m. Could no longer control my bowels (may need to wear adult diapers).	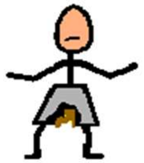	**1**	**2**	3	**4**	**5**
Comments						
n. Lived permanently in a nursing home.	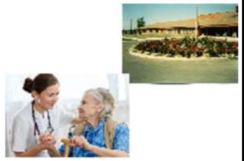	**1**	**2**	3	**4**	**5**

Graphic Values History

**What if I . . .**		**Definitely do not want treatment that prolongs life**	**May not want treatment that prolongs life**	**Uncertain**	**May want treatment that prolongs life**	**Definitely want treatment that prolongs life**
Comments						
o. Was confined to bed.	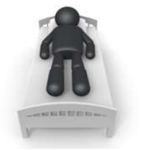	**1**	**2**	3	**4**	**5**
Comments						
p. Lived **permanently** in a Critical Care Unit.	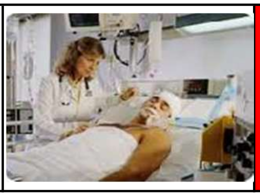	**1**	**2**	3	**4**	**5**
Comments						
q. Other.		**1**	**2**	3	**4**	**5**
Comments						

6. How do I weigh my chances of survival? ‡
People look at the pros and cons of medical treatments in very personal ways, which is why some people may choose a treatment that others refuse. All treatments carry risks. No one can predict the outcome in every case.

How much would you be willing to endure if treatment was likely to save your life, but also likely to leave you in poorer health than before you became ill?

Imagine: You are seriously ill. Doctors are recommending treatment that has harsh side effects, such as severe pain, nausea, vomiting, or weakness that could last for months. Treatment would allow you to recover from this illness, **but your overall condition would probably be somewhat worse than before you became ill (i.e., recovery but a decline in condition).** Refusal of treatment would result in death.

Question: Would I be willing to endure these side effects if the chance that I recovered was:

	Definitely not	Probably not	Uncertain	Probably would	Definitely would
a. **High:** over 80% chance I would recover to somewhat poorer health than before I got sick	1	2	3	4	5

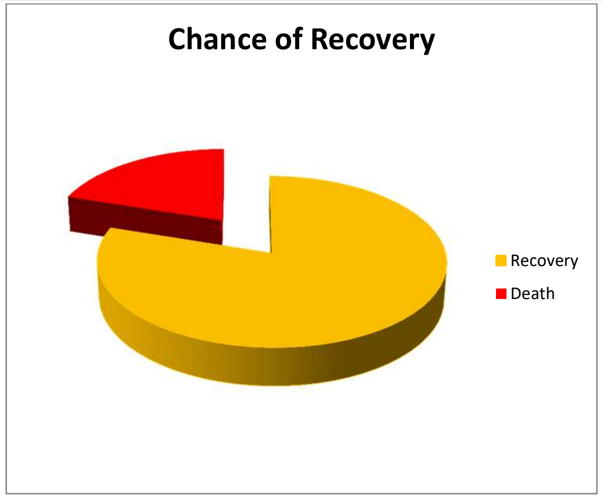

Comments:

You JJ, et al. BMJ Supportive & Palliative Care 2022; 12:e777–e784. doi: 10.1136/bmjspcare-2018-001698

Graphic Values History

	Definitely not	Probably not	Uncertain	Probably would	Definitely would
b. **Moderate:** 50% chance I would recover to somewhat poorer health than before I got sick	**1**	**2**	3	**4**	**5**

Comments

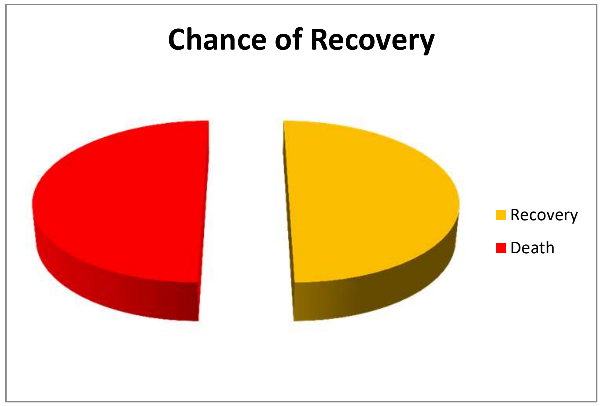

You JJ, *et al. BMJ Supportive & Palliative Care* 2022; 12:e777–e784. doi: 10.1136/bmjspcare-2018-001698

Graphic Values History

	**Definitely not**	**Probably not**	**Uncertain**	**Probably would**	**Definitely would**
c. **Low:** 20% chance I would recover to somewhat poorer health than before I got sick	**1**	**2**	3	**4**	**5**

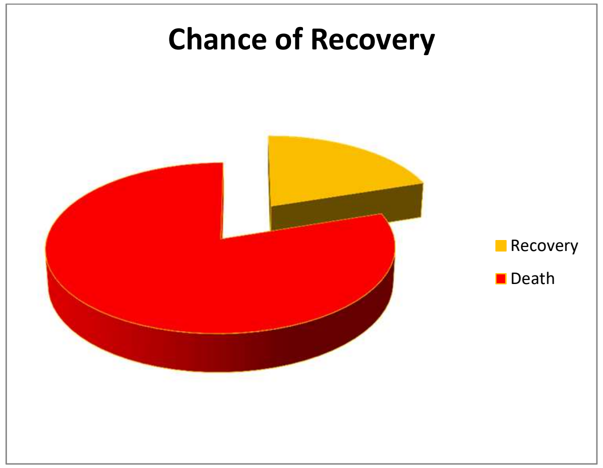

Comments:

You JJ, *et al. BMJ Supportive & Palliative Care* 2022; 12:e777–e784. doi: 10.1136/bmjspcare-2018-001698

Graphic Values History

	**Definitely not**	**Probably not**	**Uncertain**	**Probably would**	**Definitely would**
d. Very, Very **Low:** less than 1 in 1000 or .1% chance I would recover to somewhat poorer health than before I got sick	**1**	**2**	3	**4**	**5**

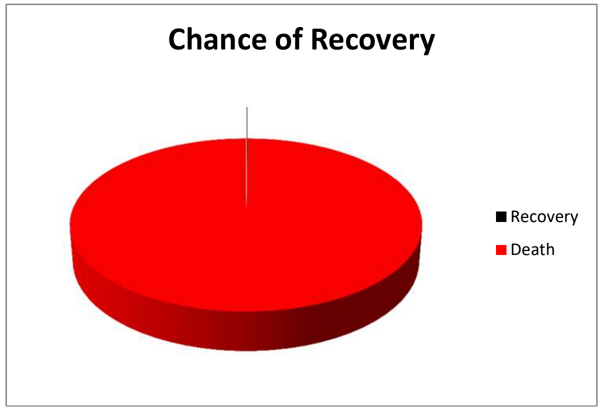

Comments:

‡Adapted from American Bar Association Commission on Law and Aging. Consumer's Tool Kit For Health Care Advance Care Planning: Tool #2. Are some conditions worse than death? Second edition, 2005

Graphic Values History

7. Impact of Decision on Others

Most of us live within relationships (e.g., family, friends). As such our decisions impact others (i.e, financially, physically, emotionally).

Instructions: Circle the most appropriate answer.

a. Do you think your family and friends will support you with the **implementation** of your wishes?	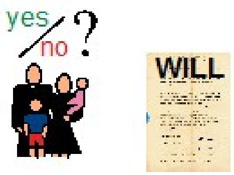	**Totally Unsupportive**	**Somewhat Unsupportive**	**Uncertain**	**Somewhat Supportive**	**Totally Supportive**
		**1**	**2**	**3**	**4**	**5**
Comments:						
b. How much do you think your decisions will impact your family and friends?	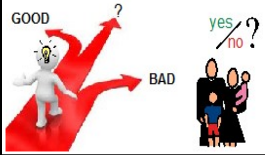	**To a Great Extent**	**Somewhat**	**Uncertain**	**Very Little**	**Not At All**
		**1**	**2**	**3**	**4**	**5**
Comments:						

You JJ, *et al. BMJ Supportive & Palliative Care* 2022; 12:e777–e784. doi: 10.1136/bmjspcare-2018-001698

Graphic Values History

c. How concerned are you about being a:		Greatly Concerned	Concerned	Uncertain	Not Concerned	Not at all Concerned
i. Financial burden?	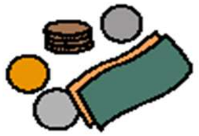	1	2	3	4	5
Comments:						
ii. Physical burden?	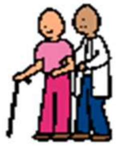	1	2	3	4	5
Comments:						
iii. Emotional burden?	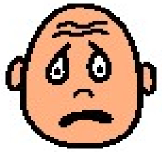	1	2	3	4	5
Comments:						

You JJ, *et al. BMJ Supportive & Palliative Care* 2022; 12:e777–e784. doi: 10.1136/bmjspcare-2018-001698

Graphic Values History

8. Religious / Spiritual / Cultural Beliefs

Religious / Spiritual beliefs play an important role in end of life decision making for some people. For others, Religious / Spiritual beliefs are not important at this time.

Instructions: Please circle the most appropriate answer .

**a. What importance do religious / spiritual beliefs play in guiding your medical treatment?**	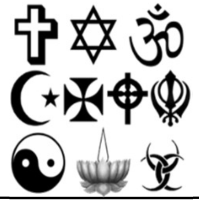	**Not Important**	**Of little importance**	**Uncertain**	**Important**	**Very Important**
		**1**	**2**	3	**4**	**5**

Please describe **religious/spiritual** beliefs you wish respected.

**b. What importance do cultural practices play in guiding your medical treatment?**	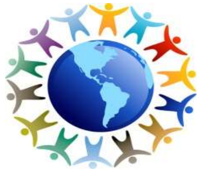	**Not Important**	**Of little importance**	**Uncertain**	**Important**	**Very Important**
		**1**	**2**	3	**4**	**5**

Please describe **cultural practices** you wish respected.

You JJ, *et al. BMJ Supportive & Palliative Care* 2022; 12:e777–e784. doi: 10.1136/bmjspcare-2018-001698

c. The thing that concerns, scares or worries you the most is:

d. Describe any other values or beliefs you have regarding your medical care:

Statement:
This document represents my values. I want my substitute decision-maker to use my values when making decisions about my health care.

Date: ____

Signature:_______________________________________

Witness #1 ____

Witness #2 ____

Demographics

1. What is your age?
   - □ < 20 yrs
   - □ 21 - 30 yrs
   - □ 31 - 40 yrs
   - □ 41 - 50 yrs
   - □ 51 - 60 yrs
   - □ 61 - 70 yrs
   - □ 71+ yrs

2. Please provide a brief description of your **current health condition**:

3. How long have you been living with your current condition(s)?
   - □ < 1 yr
   - □ 1 - 5 yrs
   - □ 6 - 10 yrs
   - □ 11 - 19 yrs
   - □ 20 +

You JJ, *et al. BMJ Supportive & Palliative Care* 2022; 12:e777–e784. doi: 10.1136/bmjspcare-2018-001698

4. What is the highest level of education you completed?

□ Nursery school to Gr. 8
□ Some high school, no diploma
□ High school graduate, diploma or equivalent
□ Some college credit, no degree
□ Trade/technical/vocational training
□ Bachelor's degree
□ Master's degree
□ Professional degree
□ Doctorate degree

5. Over the last 6 months, did you find it difficult to pay for all of the following: prescription medications, home heating, adequate food or home care, rent/mortgage payr

□ Yes
□ No

Appendix 3. Revisions to draft Short GVHT in response to review by 20 beta-testers

**Formatting changes**
1. Comments boxes throughout document have been increased in size to allow more space to write comments.
2. S1. & S3 Footnote indicators † ‡ changed to numbers. One person became upset at what was perceived as a religious symbol.
3. S1. “Definition” removed to reduce the number of bolded words
**Wording changes**
1. P3. Intro – Added new section, titled **More than one change**: “This document asks questions about one change (or altered condition of health) at a time. Please try to weigh each scenario by itself. … When several changes happen at the same time, or when one change is quickly followed by another change, it can be much more difficult to adjust. If you wish, you can use the comments section to discuss the impact of several changes happening at the same time.”
2. P3. Intro. – “While patient autonomy is extremely important, it is **not absolute**.” Changed to “While patient autonomy is extremely important, health care professionals cannot be forced to comply with wishes that are illegal.”
3. P3. How to use this tool – simplified language: “It can **complement** any other statement of treatment wishes.” Changed to “It can be used along with any other statement of treatment wishes.”
4. P3. How to use this tool – simplified language: “In the event the patient is found to be **incapable** of making treatment decisions” **changed to** “If the patient is unable to make treatment decisions… .”
5. P3. Intro. – Added definition of Advance Care Planning in footnote

6. P3. Intro. – Added definition of Autonomy in footnote
7. S1. Qg. -- **Added** "or friends." Now reads: "Unable to recognize family **or friends**."
8. S3. Intro. – "This worksheet looks at **such situations**" **changed to;** "This worksheet looks at some of these situations."
9. S3. Q.g. – **Added** in brackets "Food and fluid are provided by a tube into the stomach. I do not taste foods."
10. S.3. Q.i. – **Added** in brackets "The machine blows oxygen into the lungs through a tube in the mouth or throat."
11. S4. Qa. – "Do you think your family and friends will support you with the **implementation** of your wishes?" **changed to** "Do you think your family and friends will support you with the **carrying out** of your wishes?"

Appendix 4. Short Graphic Values History Tool (GVHT); patient version

What's important to me

a graphic values history tool (short version)

Questions for Patients

Peter Allatt, Clinical Ethicist

Version Date: July 04, 2015

What are values?

What are values? Values are the things that matter to you -- what you feel is important in life.

Values and Medical Decisions

In health care, your wishes for treatment will depend on your values. Some people, for example, value independence, and would not want treatment that prolongs life if they could no longer live independently. Others, who are less concerned about independence, may feel that their ability to communicate with family and friends is most important. Because we all have different values, some people will decide to have a treatment that others refuse.

"What's important to me" asks questions to help you think about your values.

Advance Care Planning

Most people want to make their own health care decisions.[1] Because of illness or injury, many people will unfortunately lose the ability to make these decisions. If this were to happen to you, a substitute decision maker[2] would be asked to make decisions for you.

You can decide what you want now, while you are capable of making decisions, and tell your substitute decision maker. Telling your substitute decision maker what you do and do not want is a way of extending your control over the health care you will receive in the future. We call this advance care planning.

Advance care planning[3] is a back-and-forth process of making choices for your care in the future. Everyone, from healthy people to terminally ill patients, can benefit from advance care planning. The most important part of the process is the discussion with your substitute decision maker. You may also find it helpful to discuss your plans with a trusted health care professional.

You should review your choices every once in a while, and particularly if your health changes, or if you change your mind about what you want done and not done.

Values, not Symptoms
"What's important to me" puts the focus on patient values. Many conditions can cause symptoms such as shortness of breath or constipation. What matters to patients is how they feel about the symptom, and what they wish to be done. If you had such a symptom and there was no chance of recovery, would your quality of life be acceptable to you?

Completing the document
Please take the time you need to complete this document. Every comment you make gives valuable information to your substitute decision maker, and will help guide the discussion that follows. You don't have to answer every question, but try to answer as many questions as you can.

More than one change
This document asks questions about one change (or symptom) at a time. Please try to weigh each scenario by itself.

When several changes happen at the same time, or when one change is quickly followed by another change, it can be much more difficult to adjust. If you wish, you can use the comments section to discuss the impact of several changes happening at the same time.

Overview
This tool has 5 sections:
1. Quality of life
2. Value trade-offs
3. Are some conditions worse than death?
4. Impact of decisions on others
5. Religious / spiritual / cultural beliefs

How to use this tool

This tool can be used in a number of ways. It can be used:

1. To begin advance care planning, as a way of identifying the values you wish to guide treatment decisions. It can be used alone, or along with any other statement of treatment wishes (e.g., living will).
2. When you've finished advance care planning, to check that your treatment decisions are in keeping with your values.
3. When you are faced with immediate medical decisions the document can help you decide what is important to you.
4. To start conversations between you and your substitute decision maker, or between you and your doctors and nurses. Health care professionals can use this tool to spell out what you hold important as you or your SDM make treatment decisions.

Autonomy and the Law

While patient autonomy[1] is important, health care professionals cannot be forced to comply with wishes that are illegal.

1. **Autonomy:** the right of a person to make decisions for themselves.
2. **Substitute Decision Maker:** someone who makes decisions on your behalf if you become incapable of making them.
3. **Advance Care Planning:** choices made by a capable person for the future when they become incapable.

1. Quality of Life

Definition
Quality of life - The degree of satisfaction, comfort and enjoyment in life, what makes life worth living.

Background
What gives you quality of life is a personal decision. There are no right or wrong answers. For example, one person may say "being unable to walk, could only get around by wheelchair" is "somewhat acceptable" but, "to be unable to understand what others say or to communicate my thoughts" is "totally unacceptable". Others may find both "somewhat unacceptable".

This section looks at the value of independence in **YOUR** quality of life. Consider whether your quality of life would be acceptable if you found yourself in the following circumstances, **with no reasonable chance of improvement**.

Instructions: Circle the most appropriate answer.

You JJ, *et al. BMJ Supportive & Palliative Care* 2022; 12:e777–e784. doi: 10.1136/bmjspcare-2018-001698

What's important to me

a. What would I say about my quality of life if I were **unable to walk, could only get around by wheelchair?** 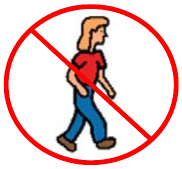			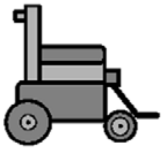		
**Totally Unacceptable 1**	**Somewhat Unacceptable 2**	**Neutral 3**	**Somewhat Acceptable 4**	**Totally Acceptable 5**	**Unsure/ Don't Know**
**Comments:**					
b. What would I say about my quality of life if I were **unable to feed myself?** 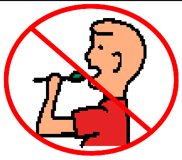			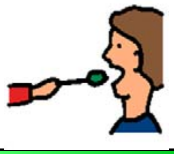		
**Totally Unacceptable 1**	**Somewhat Unacceptable 2**	**Neutral 3**	**Somewhat Acceptable 4**	**Totally Acceptable 5**	**Unsure/ Don't Know**
**Comments:**					
c. What would I say about my quality of life if I were **unable to wash myself?** 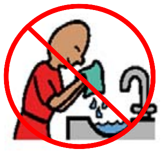			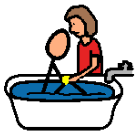		
**Totally Unacceptable 1**	**Somewhat Unacceptable 2**	**Neutral 3**	**Somewhat Acceptable 4**	**Totally Acceptable 5**	**Unsure/ Don't Know**
**Comments:**					

You JJ, *et al. BMJ Supportive & Palliative Care* 2022; 12:e777–e784. doi: 10.1136/bmjspcare-2018-001698

What's important to me

d. What would I say about my quality of life if I were **unable to control my bowels or bladder (may need to wear adult diapers)?**

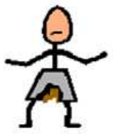

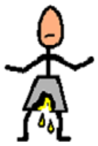

Totally Unacceptable 1	Somewhat Unacceptable 2	Neutral 3	Somewhat Acceptable 4	Totally Acceptable 5	Unsure/ Don't Know
**Comments:**					

e. What would I say about my quality of life if I were **unable to understand what others say or to communicate my thoughts?**

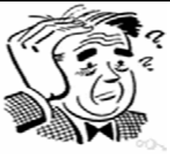

Totally Unacceptable 1	Somewhat Unacceptable 2	Neutral 3	Somewhat Acceptable 4	Totally Acceptable 5	Unsure/ Don't Know
**Comments:**					

f. What would I say about my quality of life if I were **unable to make my own decisions?**

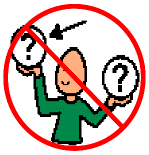

Totally Unacceptable 1	Somewhat Unacceptable 2	Neutral 3	Somewhat Acceptable 4	Totally Acceptable 5	Unsure/ Don't Know
**Comments:**					

What's important to me

g. What would I say about my quality of life if I were **unable to recognize family/friends?**

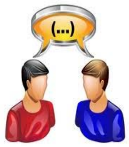

Totally Unacceptable 1	Somewhat Unacceptable 2	Neutral 3	Somewhat Acceptable 4	Totally Acceptable 5	Unsure/ Don't Know
**Comments:**					

h. What would I say about my quality of life if I were **unable to get outside and must spend all day at home?**

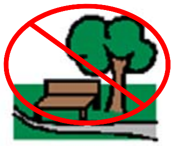

Totally Unacceptable 1	Somewhat Unacceptable 2	Neutral 3	Somewhat Acceptable 4	Totally Acceptable 5	Unsure/ Don't Know
**Comments:**					

i. What would I say about my quality of life if I were **confined to bed and/or needing someone to take care of me 24 hours a day?**

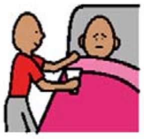

Totally Unacceptable 1	Somewhat Unacceptable 2	Neutral 3	Somewhat Acceptable 4	Totally Acceptable 5	Unsure/ Don't Know
**Comments:**					

You JJ, *et al. BMJ Supportive & Palliative Care* 2022; 12:e777–e784. doi: 10.1136/bmjspcare-2018-001698

What's important to me

j. What would I say about my quality of life if I were **unable to return to the place where I lived before I got sick?**

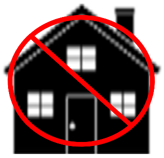

Totally Unacceptable 1	Somewhat Unacceptable 2	Neutral 3	Somewhat Acceptable 4	Totally Acceptable 5	Unsure/ Don't Know
**Comments:**					

k. What would I say about my quality of life if I were **needing to live permanently in a nursing home?**

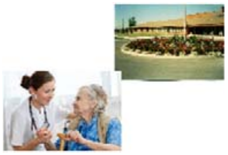

Totally Unacceptable 1	Somewhat Unacceptable 2	Neutral 3	Somewhat Acceptable 4	Totally Acceptable 5	Unsure/ Don't Know
**Comments:**					

l. What would I say about my quality of life if I were **needing to live in a hospital?**

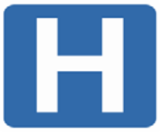

Totally Unacceptable 1	Somewhat Unacceptable 2	Neutral 3	Somewhat Acceptable 4	Totally Acceptable 5	Unsure/ Don't Know
**Comments:**					

You JJ, *et al. BMJ Supportive & Palliative Care* 2022; 12:e777–e784. doi: 10.1136/bmjspcare-2018-001698

m. Do you have other issues or concerns about your quality of life?

Write your issue here, and rate its importance.

Totally Unacceptable 1	Somewhat Unacceptable 2	Neutral 3	Somewhat Acceptable 4	Totally Acceptable 5	Unsure/ Don't Know
**Comments:**					

You JJ, *et al. BMJ Supportive & Palliative Care* 2022; 12:e777–e784. doi: 10.1136/bmjspcare-2018-001698

2. Exploring value trade-offs

Definition

Trade-off - A situation where you must choose between two things of equal value. You may have to lose some of the first thing to gain more of the second thing.

Background

When two important values compete, patients may have to chose one over the other. These choices are personal, and can be difficult to make.

Instructions: Place an **"X" on the line** to show which value is most important to you.

a. What would I say is more important -- **How well I live** or **how long I live?**

**How well I live**<br>Quality of Life	**Equally important**	**How long I live**<br>Quantity of Life

Comments:

You JJ, *et al. BMJ Supportive & Palliative Care* 2022; 12:e777–e784. doi: 10.1136/bmjspcare-2018-001698

What's important to me

3. Are some conditions worse than death? [1]

Many treatments keep people alive even if there is no reasonable chance that the treatment will reverse or improve their condition. This section looks at some of these situations. In each situation, ask yourself: how acceptable would it be to continue treatment that prolongs my life, even if the treatment would **NOT reverse or improve my condition?**

Instructions: Circle the most appropriate answer.

a. What if **I was constantly short of breath**.

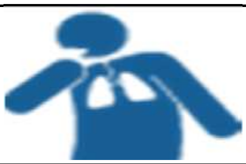

Totally Unacceptable 1	Somewhat Unacceptable 2	Neutral 3	Somewhat Acceptable 4	Totally Acceptable 5	Unsure/ Don't Know
**Comments:**					

b. What if **I was constantly thirsty**.

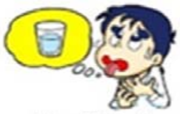

Totally Unacceptable 1	Somewhat Unacceptable 2	Neutral 3	Somewhat Acceptable 4	Totally Acceptable 5	Unsure/ Don't Know
**Comments:**					

1. Adapted from American Bar Association Commission on Law and Aging. Consumer's Tool Kit For Health Care Advance Care Planning: Tool #2. Are some conditions worse than death? Second edition, 2005

What's important to me

c. What if **I was in severe discomfort (such as nausea, diarrhea, constipation) most of the time**.

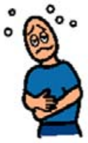
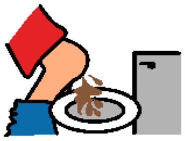

Totally Unacceptable 1	Somewhat Unacceptable 2	Neutral 3	Somewhat Acceptable 4	Totally Acceptable 5	Unsure/ Don't Know
**Comments:**					

d. What if **I was unable to understand what others say or communicate my thoughts**.

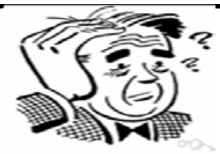

Totally Unacceptable 1	Somewhat Unacceptable 2	Neutral 3	Somewhat Acceptable 4	Totally Acceptable 5	Unsure/ Don't Know
**Comments:**					

e. What if I was unable to make my own decisions.

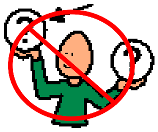

Totally Unacceptable 1	Somewhat Unacceptable 2	Neutral 3	Somewhat Acceptable 4	Totally Acceptable 5	Unsure/ Don't Know
**Comments:**					

You JJ, *et al. BMJ Supportive & Palliative Care* 2022; 12:e777–e784. doi: 10.1136/bmjspcare-2018-001698

What's important to me

f. What if **I could no longer recognize or talk with family or friends.**

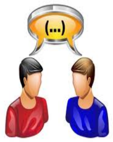

Totally Unacceptable 1	Somewhat Unacceptable 2	Neutral 3	Somewhat Acceptable 4	Totally Acceptable 5	Unsure/ Don't Know
**Comments:**					

g. What if I **needed a feeding tube to stay alive. (Food and fluid are provided by a tube into the stomach. I do not taste foods.)**

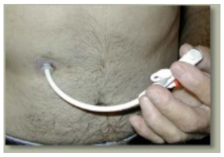

Totally Unacceptable 1	Somewhat Unacceptable 2	Neutral 3	Somewhat Acceptable 4	Totally Acceptable 5	Unsure/ Don't Know
**Comments:**					

h. What if I **needed dialysis (to be connected to a machine 4 - 6 hours per treatment, 3 treatments per week. Treatments clean the blood and remove fluid).**

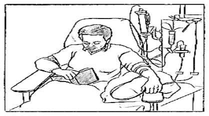

Totally Unacceptable 1	Somewhat Unacceptable 2	Neutral 3	Somewhat Acceptable 4	Totally Acceptable 5	Unsure/ Don't Know
**Comments:**					

What's important to me

i. What if I **needed to be connected to a machine 24 hours a day in order to breathe. (The machine blows oxygen into the lungs through a tube in the mouth or throat.)**

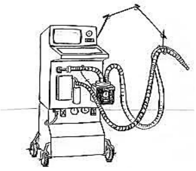

Totally Unacceptable 1	Somewhat Unacceptable 2	Neutral 3	Somewhat Acceptable 4	Totally Acceptable 5	Unsure/ Don't Know
Comments:					

j. What if I **needed to live in Critical Care Unit for the rest of my life (only place care can be provided).**

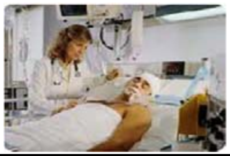

Totally Unacceptable 1	Somewhat Unacceptable 2	Neutral 3	Somewhat Acceptable 4	Totally Acceptable 5	Unsure/ Don't Know
Comments:					

k. What if I **was in severe untreatable physical pain most of the time.**

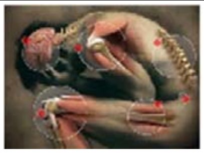

Totally Unacceptable 1	Somewhat Unacceptable 2	Neutral 3	Somewhat Acceptable 4	Totally Acceptable 5	Unsure/ Don't Know
Comments:					

You JJ, *et al. BMJ Supportive & Palliative Care* 2022; 12:e777–e784. doi: 10.1136/bmjspcare-2018-001698

What's important to me

l. What other conditions would you consider worse than death?
Please explain.

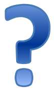

Totally Unacceptable 1	Somewhat Unacceptable 2	Neutral 3	Somewhat Acceptable 4	Totally Acceptable 5	Unsure/ Don't Know
**Comments:**					

What's important to me

4. Impact of Decision on Others
Relationships with family and friends are important to most of us, and our decisions may impact others (i.e, financially, physically, emotionally).

Instructions: Circle the most appropriate answer.

a. Do you think your family and friends will support you in **carrying out** your wishes?

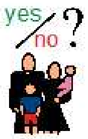

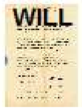

Totally Unsupportive 1	Somewhat Unsupportive 2	Neutral 3	Somewhat Supportive 4	Totally Supportive 5	Unsure	Undecided
**Comments:**						

b. Are you concerned that you may be a burden?

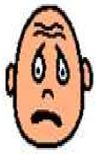
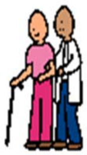
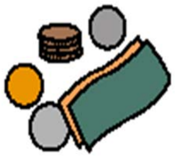

Greatly Concerned 1	Concerned 2	Neutral 3	Not Concerned 4	Not at all Concerned 5	Unsure	Undecided
**Comments:**						

You JJ, *et al. BMJ Supportive & Palliative Care* 2022; 12:e777–e784. doi: 10.1136/bmjspcare-2018-001698

What's important to me

5. Religious / Spiritual / Cultural Beliefs

Religious, spiritual, and cultural beliefs play an important role in end of life decision making for some people. For others, these beliefs are not important at this time.

Instructions: Please circle the most appropriate answer .

a. What importance do religious / spiritual beliefs play in guiding your medical treatment?

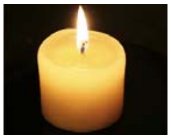

Not Important 1	Of Little Importance 2	Neutral 3	Important 4	Very Important 5	Unsure	Undecided

Please describe **religious/spiritua**l beliefs you wish respected.

b. What importance does culture play in guiding your medical treatment?

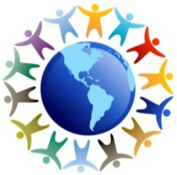

Not Important 1	Of Little Importance 2	Neutral 3	Importance 4	Very Important 5	Unsure	Undecided

Please describe **cultural practices** you wish respected.

You JJ, *et al. BMJ Supportive & Palliative Care* 2022; 12:e777–e784. doi: 10.1136/bmjspcare-2018-001698

c. The thing that concerns, scares or worries you the most is:

d. Describe any other values or beliefs you have regarding your medical care:

Statement:
This document represents my values. I want my substitute decision-maker to use my values when making decisions about my health care.

Date: ____

Signature:_______________________________________

Witness #1 ____

Witness #2 ____

Appendix 5. Decisional Conflict Scale (10-item low literacy version)

Preference for use or non-use of life-sustaining treatments

An important part of health care involves caring for people who are very sick. Many patients are too unwell to participate in decisions about their medical care when they become very sick. Hospital doctors will often talk to patients early in their hospital stay about what treatments the patient would want if his or her heart stopped. Some may find this topic alarming if it is the first time they have ever thought about it. Others may have thought about resuscitation in the past and have talked with their family and their doctors about their wishes.

At this point in time, if life supports were needed to keep you alive, which option would you prefer for your care? Please choose (√) one.

❑	Use machines and all possible measures including resuscitation (CPR) with a focus on keeping me alive at all costs.
❑	Use machines and all possible measures with a focus on keeping me alive but if my heart stops, no resuscitation (CPR).
❑	Use machines only in the short term to see if I will get better but if the illness is prolonged, change focus to comfort measures only. If my heart stops, no resuscitation (CPR).
❑	Use full medical care to prolong my life but if my heart or my breathing stops, no resuscitation (CPR) or breathing machines.
❑	Use comfort measures only with a focus on improving my quality of life and comfort. Allow natural death and no artificial prolongation of life and no resuscitation (CPR).
❑	Unsure

Decisional Conflict Scale (10-item low literacy version)

Considering the option you prefer, please answer the following questions:

	Yes [0]	Unsure [2]	No [4]
1. Do you know which options are available?	☐	☐	☐
2. Do you know the benefits of each option?	☐	☐	☐
3. Do you know the risks and side effects of each option?	☐	☐	☐
4. Are you clear about which benefits matter most to you?	☐	☐	☐
5. Are you clear about which risks and side effects matter most to you?	☐	☐	☐
6. Do you have enough support from others to make a choice?	☐	☐	☐
7. Are you choosing without pressure from others?	☐	☐	☐
8. Do you have enough advice to make a choice?	☐	☐	☐
9. Are you clear about the best choice for you?	☐	☐	☐
10. Do you feel sure about what to choose?	☐	☐	☐

Appendix 6. Values domain of the Advance Care Planning (ACP) Engagement Survey (ACP-Values)

Introduction	**In this survey, we will ask about your experiences and opinions. We may ask about things that you have already done, or have not thought about at all. Just answer as honestly as you can.**<br>**In this questionnaire we will be asking you about deciding what matters most in life.**<br>**Some questions will ask you about a MEDICAL DECISION MAKER, what we mean by this term is someone who will make decisions for you if you become unable to make your own decisions.**

Now we want to talk about how some people feel about their quality of life. For instance, how they want to live, and how they do not want to live. Some people think that all health situations or experiences, such as being in a coma or not being able to get out of bed, are worth living through. Other people think that some health situations or experiences would make their life not worth living.

Please give us your honest opinions. There are no right or wrong answers.

THOUGHT ABOUT IT (HEALTH SITUATIONS)

The following questions ask about how much you have thought about something. *[Read options.]*

**How much have you thought about…**	Never	Once or twice	A few times	Several times	A lot	Not Sure	Ref.
1. Whether or not certain health situations would make your life not worth living? (PE_S2A_T1)	1	2	3	4	5	8	9
2. Talking with your MEDICAL DECISION MAKER about whether or not certain health situations would make your life not worth living? (PE_S2A_T2)	1	2	3	4	5	8	9
3. Talking with your DOCTOR about whether or not certain health situations would make your life not worth living? (PE_S2A_T3)	1	2	3	4	5	8	9
4. Talking with your other FAMILY and FRIENDS about whether or not certain health situations would make your life not worth living? (PE_S2A_T4)	1	2	3	4	5	8	9

SELF-EFFICACY (HEALTH SITUATIONS)

The next three questions ask about how confident you are to actually talk to someone about your medical wishes. *[Read options.]*

**How confident are you that today you could….**	Not at all	A little	Somewhat	Fairly	Extremely	Not Sure	Ref.
5. Talk with your MEDICAL DECISION MAKER about whether or not certain health situations would make your life not worth living? (PE_S2A_SE1)	1	2	3	4	5	8	9
6. Talk with your DOCTOR about whether or not certain health situations would make your life not worth living? (PE_S2A_SE2)	1	2	3	4	5	8	9
7. Talk with your other FAMILY and FRIENDS about whether or not certain health situations would make your life not worth living? (PE_S2A_SE3)	1	2	3	4	5	8	9

DECISIONS MADE (HEALTH SITUATIONS)

The following questions are about decisions you may have already made. These are things you may have decided in your own mind, even if you haven't talked with anyone about it yet.

Again, health situations can be such things as being in a coma, or not being able to get out of bed.

8. How ready are you to decide whether or not certain health situations would make your life not worth living? (PE_S2A_SIT_RDY)<br>1 ☐ I have never thought about it<br>2 ☐ I have thought about it, but I am not ready to do it<br>3 ☐ I am thinking about doing it in the next 6 months<br>4 ☐ I am definitely planning to do it in the next 30 days<br>5 ☐ I have already done it	8☐ Not Sure<br>9☐ Refused

ACTIONS (HEALTH SITUATIONS)

9. How ready are you to talk to your DECISION MAKER about whether or not certain health situations would make your life not worth living? (PE_S2A_TELLDM_RDY)<br>1 ☐ I have never thought about it<br>2 ☐ I have thought about it, but I am not ready to do it<br>3 ☐ I am thinking about doing it in the next 6 months<br>4 ☐ I am definitely planning to do it in the next 30 days<br>5 ☐ I have already done it	8☐ Not Sure<br>9☐ Refused

10. How ready are you to talk to your DOCTOR about whether or not certain health situations would make your life not worth living? (PE_S2A_TELLDR_RDY)<br>1 ☐ I have never thought about it<br>2 ☐ I have thought about it, but I am not ready to do it<br>3 ☐ I am thinking about doing it in the next few visits<br>4 ☐ I am definitely planning to do it at the next visit<br>5 ☐ I have already done it	8☐ Not Sure<br>9☐ Refused

Now I am going to ask you about talking to <u>other</u> family and friends, besides your decision maker.

11. How ready are you to talk to your other FAMILY and FRIENDS about whether OR NOT certain health situations would make your life not worth living? (PE_S4_SIT_RDY)<br>1 ☐ I have never thought about it<br>2 ☐ I have thought about it, but I am not ready to do it<br>3 ☐ I am thinking about doing it in the next 6 months<br>4 ☐ I am definitely planning to do it in the next 30 days<br>5 ☐ I have already done it	8☐ Not Sure<br>9☐ Refused

We are switching topics now. The previous questions were about how people would or would not want to live.

The following questions are about specific medical treatments that people may or may never want if they were very sick or at the end of their life. For instance, some people know they would want to be on a breathing machine. Other people know they would never want to be on a breathing machine. Please give us your honest opinions to the following questions about medical treatments. There are no right or wrong answers.

THOUGHT ABOUT IT **(CARE AT EOL)**

The following questions ask about how much you have thought about any of the following. *[Read options.]*

**How much have you thought about…**	Never	Once or twice	A few times	Several times	A lot	Not Sure	Ref.
12. The care you would want if you were very sick or near the end of life? (PE_S2B_T1)	1	2	3	4	5	8	9
13. Talking with your MEDICAL DECISION MAKER about the care you would want if you were very sick or near the end of life? (PE_S2B_T2)	1	2	3	4	5	8	9
14. Talking with your DOCTOR about the care you would want if you were very sick or near the end of life? (PE_S2B_T3)	1	2	3	4	5	8	9
15. Talking with your other FAMILY and FRIENDS about the care you would want if you were very sick or near the end of life? (PE_S2B_T4)	1	2	3	4	5	8	9

SELF-EFFICACY **(CARE AT EOL)**

The next three questions ask about how confident you are to actually talk about your medical wishes. *[Read options.]*

**How confident are you that today you could….**	Not at all	A little	Somewhat	Fairly	Extremely	Not Sure	Ref.
16. Talk with your MEDICAL DECISION MAKER about the care you would want if you were very sick or near the end of life? (PE_S2B_SE1)	1	2	3	4	5	8	9
17. Talk with your DOCTOR about the care you would want if you were very sick or near the end of life? (PE_S2B_SE2)	1	2	3	4	5	8	9
18. Talk with your other FAMILY and FRIENDS about the care you would want if you were very sick or near the end of life? (PE_S2B_SE3)	1	2	3	4	5	8	9

DECISIONS MADE **(CARE AT EOL)**

The following questions are about decisions you may have already made. These are things you may have decided in your own mind, even if you haven't talked with anyone about it yet.

19. How ready are you to decide on the medical care you would want if you were very sick or near the end of life? (PE_S2_CARE_RDY)
    - 1 ☐ I have never thought about it
    - 2 ☐ I have thought about it, but I am not ready to do it
    - 3 ☐ I am thinking about doing it in the next 6 months
    - 4 ☐ I am definitely planning to do it in the next 30 days
    - 5 ☐ I have already done it

    8 ☐ Not Sure
    9 ☐ Refused

ACTIONS **(CARE AT EOL)**

20. How ready are you to talk to your DECISION MAKER about the kind of medical care you would want if you were very sick or near the end of life? (PE_S2B_TELLDM_READY)
    - 1 ☐ I have never thought about it
    - 2 ☐ I have thought about it, but I am not ready to do it
    - 3 ☐ I am thinking about doing it in the next 6 months
    - 4 ☐ I am definitely planning to do it in the next 30 days
    - 5 ☐ I have already done it

    8 ☐ Not Sure
    9 ☐ Refused

21. How ready are you to talk to your DOCTOR about the kind of medical care you would want if you were very sick or near the end of life? (PE_S2B_TELLDR_RDY)
    - 1 ☐ I have never thought about it
    - 2 ☐ I have thought about it, but I am not ready to do it
    - 3 ☐ I am thinking about doing it in the next few visits
    - 4 ☐ I am definitely planning to do it at the next visit
    - 5 ☐ I have already done it

    8 ☐ Not Sure
    9 ☐ Refused

Now I am going to ask you a question about talking to <u>other</u> family and friends, besides your decision maker.

22. How ready are you to talk to your other FAMILY and FRIENDS about the kind of medical care you would want if you were very sick or near the end of life? (PE_S4_CARE_RDY)
    - 1 ☐ I have never thought about it
    - 2 ☐ I have thought about it, but I am not ready to do it
    - 3 ☐ I am thinking about doing it in the next 6 months
    - 4 ☐ I am definitely planning to do it in the next 30 days
    - 5 ☐ I have already done it

    8 ☐ Not Sure
    9 ☐ Refused
